# A pragmatic, randomized, controlled study evaluating the impact of access to smoking cessation pharmacotherapy coverage on the proportion of successful quitters in a Canadian population of smokers motivated to quit (ACCESSATION)

**DOI:** 10.1186/1471-2458-14-433

**Published:** 2014-05-07

**Authors:** Peter Selby, Gerald Brosky, Paul Oh, Vincent Raymond, Carmen Arteaga, Suzanne Ranger

**Affiliations:** 1 Addictions Division, Centre for Addiction and Mental Health, Toronto, Ontario, Canada; 2 Departments of Family and Community Medicine and Psychiatry and the Dalla Lana School of Public Health, University of Toronto, Toronto, Ontario, Canada; 3 Ontario Tobacco Research Unit, Toronto, Ontario, Canada; 4 Department of Family Medicine, Dalhousie University, Halifax, Nova Scotia, Canada; 5 Cardiac Rehabilitation and Secondary Prevention Program, Toronto Rehabilitation Institute, Toronto, Canada; 6 Health Economics and Outcomes Research, Pfizer Canada Inc, Kirkland, Québec,Canada; 7 Chantix/Champix Project Statistical Lead, Pfizer Inc, New York, NY, USA; 8 Pfizer Canada Inc, Kirkland, Québec, Canada

**Keywords:** Bupropion, Clinical trial, Medications, Nicotine, Pharmacotherapy coverage, Policy, Pragmatic, Reimbursement, Smoking cessation, Varenicline

## Abstract

**Background:**

Many smokers find the cost of smoking cessation medications a barrier. Financial coverage for these medications increases utilization of pharmacotherapies. This study assesses whether financial coverage increases the proportion of successful quitters.

**Methods:**

A pragmatic, open-label, randomized, controlled trial was conducted in 58 Canadian sites between March 2009 and September 2010. Smokers (≥10 cigarettes/day) without insurance coverage who were motivated to quit within 14 days were randomized (1:1) in a blinded manner to receive either full coverage eligibility for 26 weeks or no coverage. Pharmacotherapies covered were varenicline, bupropion, or nicotine patches/gum. Investigators/subjects were unblinded to study group assignment after randomization and prior to choosing a smoking cessation method(s). All subjects received brief smoking cessation counseling. The primary outcome measure was self-reported 7-day point prevalence of abstinence (PPA) at week 26.

**Results:**

Of the 1380 randomized subjects (coverage, 696; no coverage, 684), 682 (98.0%) and 435 (63.6%), respectively, were dispensed at least one smoking cessation medication dose. The 7-day PPA at week 26 was higher in the full coverage versus no coverage group: 20.8% (*n* = 145) and 13.9% (*n* = 95), respectively; odds ratio (OR) = 1.64, 95% confidence interval (CI) 1.23–2.18; *p* = 0.001. Urine cotinine-confirmed 7-day PPA at week 26 was 15.7% (*n* = 109) and 10.1% (*n* = 69), respectively; OR = 1.68, 95% CI 1.21–2.33; *p* = 0.002. After pharmacotherapy, coverage eligibility was withdrawn from the full coverage group, continuous abstinence between weeks 26 and 52 was 6.6% (*n* = 46) and 5.6% (*n* = 38), in the full coverage and no coverage groups, respectively; OR = 1.19, 95% CI 0.76–1.87; *p* = 0.439.

**Conclusions:**

In this study, the adoption of a smoking cessation medication coverage drug policy was an effective intervention to improve 26-week quit rates in Canada. The advantages were lost once coverage was discontinued. Further study is required on the duration of coverage to prevent relapse to smoking. (clinicaltrials.gov identifier: NCT00818207; the study was sponsored by Pfizer Inc.).

## Background

Tobacco addiction is associated with one in eight deaths worldwide [1]. The efficacy of all smoking cessation medications (pharmacotherapy) i.e., varenicline, bupropion, and nicotine replacement therapies (NRTs; patch, gum, lozenge, inhaler, and nasal spray) is well established [2-6]. Nicotine replacement therapies (odds ratio [OR] = 1.89, credible interval [CrI] 95% 1.63–2.18), bupropion (OR = 1.95, CrI 95% 1.58–2.41), and varenicline (OR = 2.78, CrI 95% 2.17–3.57) all increase the odds of 12-month abstinence compared with placebo [5]. Although there are many reasons why smokers do not access pharmacotherapy, lack of insurance coverage remains a significant barrier [7]. Pharmacotherapy coverage may increase the number of quit attempts, the likelihood of pharmacotherapy utilization and adherence, and ultimately, smoking cessation.

Despite pharmacotherapies being among the most cost-effective and highly advocated interventions for coverage [8], funders have been slow to invest. Systematic reviews concluded that full coverage (versus no coverage) of pharmacotherapies could increase quit rates with low attributable incremental cost [5,9]. However, the included trials typically excluded real-world smokers who often have psychiatric and medical comorbidity, thereby limiting generalizability [10]. The few published pragmatic studies have several methodological problems such as not being randomized controlled trials (RCTs) and not including all available pharmacotherapies [9].

Based on the 10 dimensions of the PRECIS Tool to evaluate clinical trial design [11,12], this mostly pragmatic open-label RCT evaluated the effectiveness of pharmacotherapy coverage versus no coverage for 26 continuous weeks in increasing smoking cessation in ambulatory care settings in motivated adult smokers in Canada.

## Methods

### Study design and participants

This open-label RCT was conducted in 58 different sites across Canada (excepting Quebec, Alberta, and New Brunswick). Smokers were recruited by study site referrals and advertisement. Potential subjects were screened by a call centre to assess initial eligibility and drug insurance coverage. The investigator then confirmed eligibility (see Additional file 1).

Participants were adults (18–75 years) who smoked ≥10 cigarettes per day, were willing to set a quit date within 14 days following screening/randomization, had no period of abstinence >3 months in the past year, and had not attempted to quit smoking in the 30-day period before screening. Exclusion criteria were: unknown insurance status, access to unused pharmacotherapy or prescription, use of tobacco products other than cigarettes within the past month, and life-threatening illness (e.g., known or suspected cancer or other disease with a life expectancy of <1 year). Medical and psychiatric conditions were not exclusion criteria *per se*.

The study was conducted in compliance with the ethical principles originating in or derived from the Declaration of Helsinki and in compliance with all International Conference on Harmonization Good Clinical Practice Guidelines. Written informed consent for participation in the study was obtained from all participants. The final protocol, any amendments, and informed consent documentation were reviewed and approved by the Canadian Institutional Review Board (IRB) (IRB Services, Aurora, ON, Canada), or by local Canadian Ethics Committees (University of British Columbia Clinical Research Ethics Board, Vancouver, BC; Vancouver Island Health Authority Clinical Research Ethics Board, Victoria, BC; University of Manitoba Research Ethics, Winnipeg, MB; Winnipeg Clinic Ethics Committee, Winnipeg, MB; Research Ethics Board St. Joseph's Healthcare Hamilton, Hamilton, ON; University of Western Ontario Office of Research Ethics, London, ON).

### Randomization and interventions

Eligible participants were first counseled about smoking cessation and then randomly and blindly assigned (1:1; centrally to prevent gaming) at the baseline visit to receive either full coverage for 26 weeks or no coverage. Randomization was performed by the investigators using blinded lots of computer-generated randomization codes from the study biostatistician. Randomization codes were mapped to SmartPayment™ cards (drug reimbursement cards), with a distinctive color linked to the study arm to which the subject was randomized. The SmartPayment™ cards were enclosed in sealed envelopes with the randomization codes printed on the envelopes. After unblinding, the investigator and participant agreed on the pharmacological and/or non-pharmacological smoking cessation method(s) to be used. Eligible pharmacotherapies were: varenicline; bupropion; and NRT patch and gum, prescribed according to the most recent version of the respective product monograph or equivalent (see Additional file 2). A prescription had to be issued by a physician for these pharmacotherapies to be reimbursed; NRTs obtained without a prescription were not eligible for coverage, but were considered medication for statistical analysis. Participants (irrespective of allocated group) were not obliged to use a pharmacotherapy in their quit attempt. A target quit date was set within 14 days of randomization. The use of a SmartPayment™ card permitted access to either full coverage of prescribed pharmacotherapy for 26 weeks (full coverage) or a reimbursement of $5.00 per pharmacy dispensation (no coverage). This facilitated tracking of prescriptions dispensed in both groups and provided an incentive for subjects in the no coverage group to present their card at each pharmacy visit. Participants received compensation for reasonable expenses for transportation or parking for study appointments, if needed.

### Assessments and follow-up

Patient assessment, treatment recommendations, and prescriptions, as needed, were performed by physicians. Other study-related tasks were performed by trained personnel. Participants were screened at the baseline visit for demographic information, type of drug plan insurance, smoking history, Fagerström Test for Nicotine Dependence (FTND) score [13,14], comorbid medical/psychiatric history including the Columbia Suicide Severity Rating Scale (C–SSRS [15]), and concomitant medications. Brief (10-minute) smoking cessation counseling was provided to all subjects at the screening visit and at each contact, per each site’s practice. Prior to study start, all participating sites were provided brief training in current clinical recommendations/guidelines [16]. Participants made four clinic visits (weeks 1, 26, 39, and 52), and received six telephone calls (weeks 2, 13, 30, 34, 44, and 48), during which smoking status, quit attempt(s)/methods, FTND score, and abstinence status (via self-report at clinic visits) were collected. Confirmatory urinary cotinine measurements were collected, but investigators and patients were blinded to the results to mimic real-world practice (where this is not widely used). Patients were not aware that physicians were blinded to the results.

Safety evaluations included adverse event (AE) monitoring, physical examinations, and C–SSRS [15]. All observed or volunteered AEs, regardless of smoking cessation method or suspected causal relationship, were to be reported. The investigator was required to pursue information to both determine the outcome of the AE and assess whether it met criteria for a serious AE. Events were recorded up to and including 28 days after the last administration of one of the smoking cessation methods or week 26 (whichever was longer).

### Effectiveness evaluations

The primary endpoint was self-reported 7-day point prevalence of abstinence (PPA) at week 26 (end of coverage eligibility), defined as abstinence from smoking (not a single puff) for at least the preceding 7 days [17]. This pragmatic endpoint was chosen because in real-world settings there is no evidence of significant difference between estimates of smoking prevalence based on self-report versus urinary cotinine measure [18]. Whereas the primary endpoint was more pragmatic, the key secondary endpoints (self-reported 7-day PPA at week 26 and continuous abstinence rates [CAR] at weeks 26–52, confirmed by urine cotinine) were more explanatory. Other secondary endpoints were self-reported 7-day PPA at weeks 2 and 13, self-reported CAR (weeks 26–39), total number of quit attempts from randomization through week 26, and use of smoking cessation pharmacotherapies during quit attempts.

### Statistical analysis

Based on previous clinical studies of varenicline [19,20], market research, and relevant literature [21-24], quit rates in the no coverage versus full coverage group were assumed to be 15% versus 21%, respectively. A sample size of at least 676 participants per group was estimated to provide 80% power to detect a conservative difference of 6% between groups. Some consider a 2% difference clinically significant because of the estimated 50% risk reduction in smoking-related mortality associated with cessation [25]. Outcome measures were assessed using an intent-to-treat (ITT) analysis that included all randomized participants. Individuals who discontinued the study or were lost to follow-up were counted as smokers from the time of discontinuation. For calculation of CAR, participants who reported no smoking or no use of nicotine since the last contact and had a negative urine cotinine test were counted as non-smokers. Participants with missing urine cotinine test results were considered smokers.

Endpoints were analyzed using logistic regression models that included randomized group effect (full coverage versus non-coverage) as the independent variable and investigation centre as a covariate. In addition, the interaction between randomized group and centre was tested by expanding the logistic regression model to add the interaction term. In testing for the primary and key secondary endpoints, the Type-I overall error rate of 0.05 was preserved by using statistical methods that adjusted for multiple tests.

Safety data were summarized for all randomized subjects. The incidence of AEs reported during the study was summarized by randomized arm (coverage; non-coverage) and pharmacotherapy (users; non-users) for descriptive rather than comparative purposes.

## Results

### Baseline characteristics

**Figure 1:**
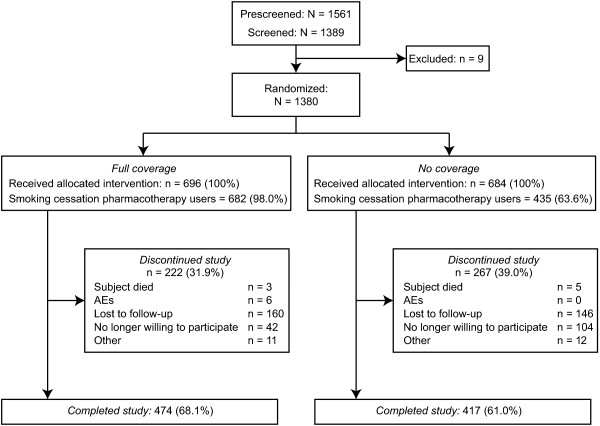
**Participant disposition **[26]**.**

Eligible participants were recruited from March 3, 2009 to September 20, 2010. Participants attended clinic visits at the time of randomization and at regular intervals for 52 weeks. Using postal codes as a proxy, participating sites were identified as being both in urban (54) and rural (4) locations. Participants were recruited by eight specialists (*n* = 115; 8.3%) and 50 generalists (*n* = 1265; 91.7%) and were referred from clinical practice (*n* = 1289; 93.4%) or advertisement (*n* = 91; 6.6%). Participant disposition is summarized in Figure 1. Baseline demographics, smoking history, and level of nicotine dependence were well balanced (Table 1). Interactions for randomization group by centre were not statistically significant (*p* > 0.4), supporting the consistency of using combined results from different centres. The primary analysis was ITT and involved all patients who were randomly assigned (696 subjects in the full coverage group and 684 subjects in the no coverage group). Participants were analysed by original group assignment.

**Table 1:** Baseline characteristics of study participants

	**Full coverage (*n*** **= 696)**	**No coverage (*n*** **= 684)**
**Demographic characteristics**		
Age, mean, years (SD)	46.5 (12.3)	46.7 (12.3)
Gender, *n* (%)		
Male	354 (50.9)	337 (49.3)
Female	342 (49.1)	347 (50.7)
Race, *n* (%)		
White	664 (95.4)	660 (96.5)
Black	11 (1.6)	6 (0.9)
Asian	15 (2.2)	13 (1.9)
Other	6 (0.9)	5 (0.7)
Educational level, years, *n* (%)		
<10	46 (6.6)	57 (8.3)
10–12	321 (46.1)	305 (44.6)
13–16	273 (39.2)	257 (37.6)
>16	56 (8.0)	61 (8.9)
Employment status, *n* (%)		
Full-time employed	298 (42.8)	297 (43.4)
Part-time employed	79 (11.4)	77 (11.3)
Not employed	319 (45.8)	308 (45.0)
Insurance type, *n* (%)		
Private insurance	51 (7.3)	53 (7.7)
Government-provided insurance	143 (20.5)	137 (20.0)
No drug insurance	484 (69.5)	479 (70.0)
Other type of insurance	18 (2.6)	14 (2.0)
Household income, *n* (%)		
<$15,000 CAD	58 (8.3)	92 (9.1)
$15,000– <$25,000 CAD	260 (37.4)	246 (36.0)
$25,000– <$50,000 CAD	203 (29.2)	218 (31.9)
$50,000– <$75,000 CAD	78 (11.2)	62 (9.1)
$75,000– <$100,000 CAD	32 (4.6)	31 (4.5)
>$100,000 CAD	22 (3.2)	17 (2.5)
Body mass index (kg/m^2^), mean (SD)	28.1 (6.3)	28.3 (6.5)
**Smoking history**		
Age at onset of smoking, years, mean (SD)	15.5 (4.5)	15.5 (4.0)
Cigarette pack years, mean (SD)	34.8 (21.8)	34.6 (21.5)
Cigarettes/day (past month), mean (range)	22.2 (9.5)	21.5 (8.3)
FTND score, mean (SD)*	5.9 (2.1)	5.9 (2.1)
Number of lifetime serious quit attempts – any method, *n* (%)		
0	93 (13.4)	64 (9.4)
1	163 (23.4)	166 (24.3)
2	143 (20.5)	148 (21.6)
≥3	297 (42.7)	306 (44.7)
**Comorbidities**^**†**^		
COPD	65 (9.3)	77 (11.3)
Lung cancer	0	2 (0.3)
Coronary heart disease	18 (2.6)	17 (2.5)
Stroke	1 (0.1)	2 (0.3)
Asthma exacerbation	76 (10.9)	66 (9.6)
Past psychiatric disorder	73 (10.5)	65 (9.5)
Current psychiatric disorder	230 (33)	213 (31.1)
Current depression	109 (15.7)	106 (15.5)
Current anxiety/anxiety disorder	66 (9.5)	61 (9.0)

CAD = Canadian dollar; COPD = chronic obstructive pulmonary disease; FTND = Fagerström Test for Nicotine Dependence; SD = standard deviation; SES = socioeconomic status.

*Scores range from 0 to 10, with higher scores indicating greater nicotine dependence. ^†^Comorbidities reported as “present” in subjects’ medical history at the time of the screening/randomization visit.

### Effectiveness of coverage of pharmacotherapy

Significantly more participants in the full coverage versus no coverage group achieved the primary endpoint of 7-day PPA at week 26 (20.8% versus 13.9%, respectively; odds ratio (OR) = 1.64, 95% confidence interval (CI) 1.23–2.18; *p* = 0.001; Table 2). Significant differences in 7-day PPA between the full coverage and no coverage groups were observed at week 2 (18.8% versus 13.3%; *p* = 0.003) and at week 13 (34.1% versus 23.7%; *p* < 0.001). The difference remained significant for urine cotinine-confirmed 7-day PPA at week 26 (15.7% versus 10.1%, respectively; OR = 1.68, 95% CI 1.21–2.33; *p* = 0.002; Table 2). After withdrawal of coverage eligibility, there was no significant difference in CAR from weeks 26 to 52 between the two groups (week 52, *n* = 6.6% versus *n* = 5.6%, respectively; OR = 1.19, 95% CI 0.76–1.87; *p* = 0.439; see Additional file 3). Similar results were noted at week 39 (7.8% versus 6.3%, respectively; OR = 1.25, 95% CI 0.82–1.90; *p* = 0.298). However, 7-day PPA data were not collected after 26 weeks and the CAR at weeks 26–52 should not be directly compared with 7-day PPA data. A post-hoc analysis showed that pharmacotherapy utilization peaked at week 2 (~90% in the full coverage group), after which it steadily decreased for the remainder of the intervention phase, reaching approximately 20% at week 26 (see Additional file 4).

**Table 2:** 7-day point prevalence of abstinence from weeks 2 to 26 and complete abstinence rate from weeks 26 to 52 (intent-to-treat)

	**Full coverage (*n * = 696), proportion of patients, %**	**No coverage (*n * = 684), proportion of patients, %**	**OR (95% CI)**	***p*-value**
**7-day PPA**				
Week 2	18.8	13.3	1.59 (1.17–2.16)	0.003
Week 13	34.1	23.7	1.72 (1.35–2.20)	<0.001
Week 26	20.8	13.9	1.64 (1.23–2.18)	0.001
Week 26 (urine cotinine-confirmed)	15.7	10.1	1.68 (1.21–2.33)	0.002
**CAR weeks 26–52**	6.6	5.6	1.19 (0.76–1.87)	0.439

CI = confidence interval; CAR = complete abstinence rate; OR = odds ratio; PPA = point prevalence of abstinence.

During the 26-week coverage eligibility period, a higher proportion of participants in the full coverage versus no coverage group made at least one quit attempt (86.9% versus 70.0%, respectively; *p* < 0.001), but the number of quit attempts were not statistically different across groups (mean 3.1 versus 2.6, respectively; *p* = 0.103), and more subjects were dispensed at least one pharmacotherapy (98.0% versus 63.6%, respectively; *p* < 0.001). The median number of daily doses dispensed per participant was higher in the full versus no coverage group, except for bupropion (70 versus 42, respectively; *p* < 0.001, for varenicline; 56 versus 18.5; *p* < 0.001, respectively, for NRTs; and 39 versus 34; *p* = 0.454, respectively, for bupropion).

In the full coverage group, varenicline was dispensed at least once for 558 participants (80.2%), nicotine patches/gum for 201 participants (28.9%), and bupropion for 60 (8.6%) participants. The corresponding figures for the no coverage group were 297 (43.4%), 126 (18.4%), and 58 (8.5%). In the full coverage group, 18.4% of participants used at least two distinct pharmacotherapies, whereas only 6.3% did so in the no coverage group. Non-pharmacological methods were used by 5.7% and 9.9%, respectively.

### Adverse events

Adverse events are listed in Table 3. Serious AEs considered by the investigator to be treatment-related were reported for two participants in the full coverage group (muscular weakness and hyperkalemia; both *n* = 1) and two in the no coverage group (violence-related symptom and depression; both *n* = 1). Results from the C–SSRS assessment were also similar between the groups and revealed a “yes” answer for suicidal ideation and/or behavior in 25 (3.7%) and 11 (2.5%) in the full coverage and no coverage groups, respectively. A suicidal attempt was reported in one subject who did not take pharmacotherapy. All-causality AEs reported for ≥5% of participants (randomized and used smoking cessation pharmacotherapy) in the full coverage and no coverage arms, respectively, were: abnormal dreams, 55 (8.1%) and 17 (3.9%); headache, 45 (6.6%) and 23 (5.3%); insomnia, 41 (6.0%) and 25 (5.7%); and nausea, 107 (15.7%) and 49 (11.3%).

**Table 3:** Reported and observed adverse events* (subjects randomized smoking and used smoking cessation medication) and Columbia Suicide Severity Rating Scale (C-SSRS) assessments^**†**^

**All-causality AEs, *n *** **(%)**	**Full coverage (*n*** **= 682)**	**No coverage (*n*** **= 435)**
Total number of AEs	1473	830
Total number of participants with AEs	451 (66.1)	271 (62.3)
Subjects who discontinued study due to AE	6 (0.9)	0
Subjects with SAEs	35 (5.1)	18 (4.1)
Subjects with severe AEs	57 (8.4)	32 (7.4)

AE = adverse event; SAE = serious adverse event.

*All observed or volunteered AEs, regardless of smoking cessation method, or selected or suspected causal relationship, were reported. For all AEs, the investigator was required to pursue and obtain information adequate both to determine the outcome of the AE and to assess whether it met the criteria for classification as a serious AE.

^†^The C–SSRS was part of the neuropsychiatric section of the medical history at screening/randomization and was administered to all subjects at given visits and at the investigator’s discretion to assist with safety reporting and evaluation of possible suicide-related AEs. A “yes” answer for suicidal ideation and/or behavior was observed in 25 (3.7%) and 11 (2.5%) in the full coverage and no coverage groups, respectively (all-randomized and treated population). A suicidal attempt was reported in one subject who did not take smoking cessation medication.

## Discussion

This is the first large-scale, pragmatic, adequately powered RCT conducted in Canadian ambulatory settings demonstrating that coverage of recommended pharmacotherapies for smoking cessation in motivated smokers significantly increases quit rates at weeks 2, 13, and 26. Using self-report and cotinine-confirmed abstinence measurements, respectively, the probability of quitting smoking at the end of the intervention in the full coverage group was 1.64 and 1.68 times larger than the probability of quitting in the no coverage group. Although there was a statistically significant difference between the self-reported and cotinine-confirmed quit rates, there was no significant difference between the groups (data not shown). The weeks 26–39 and weeks 26–52 CARs between the two groups did not differ significantly, possibly indicating the loss of this benefit upon withdrawal of pharmacotherapy coverage. Using self-reported 7-day PPA at week 26, for every 14.4 smokers who receive pharmacotherapy coverage, there will be one additional quitter. Of every 8.3 persons covered, one will experience an AE due to attempting to quit smoking, and of every 111, one will discontinue due to a serious AE. Given that smoking kills one in two people, the risks of pharmacotherapy are lower than continuing to smoke.

The results of this study could be explained by greater medication adherence and utilization in the full coverage group, and a consequent greater number of quit attempts. In the no coverage group, the larger loss to follow-up may reflect real-life experience of smokers who receive cessation counselling, but do not complete their quit attempt because of lack of coverage. However, a higher than anticipated number of subjects (63.6%) in the control group were dispensed pharmacotherapy during the intervention period. This could be explained by higher motivation of the smokers in this trial and the SmartPayment™ card reimbursement of $5.00 per pharmacy dispensation in the control group. Moreover, the study sites, although not based in academic centres, might not reflect other community ambulatory clinics. These clinics were selected to participate because of their interest in treating tobacco addiction and might have been more diligent in promoting medication to stop smoking, medication adherence, and restarting medications in those who failed to quit.

The results of our study are consistent with other studies [9,24,27]. However, our study had a greater number of quit attempts, use of pharmacotherapy, and slightly higher quit rates in both groups. This may be because our study enrolled motivated smokers and empowered the practitioner–patient dynamic by permitting tailoring of pharmacotherapy options during the coverage period. The loss of effectiveness of coverage by week 39 (13 weeks after coverage ceased) should be interpreted with caution because it is not clear if the diminished quit rates reflect the chronic relapsing nature of nicotine dependence, the erosion of the intervention effect over time [28], lack of statistical power, the intensity and frequency of the follow-up, or the stringency of the definition of a continuous quitter for a pragmatic, real-world trial. It should be noted that week-26 PPA was chosen as a pragmatic endpoint [11] and the study was not powered to detect statistical significance for CARs.

Despite the study limitations, several strengths should be highlighted. The study was community-based with minimal exclusion criteria; the majority of subjects were treated by their own physician; each smoker could choose their preferred quit method(s) (pharmacological and/or non-pharmacological); and subjects received personalized treatment rather than a standard protocol. Our study attempted to create a longer window than the usual 10–12 weeks of standard coverage during which participants could keep trying to quit and allowed for a switch to or combination with another medication. Subject recruitment was completed 5 months ahead of schedule, indicating the demand for services, easy adoption of the protocol, and feasibility.

## Conclusions

The adoption of a pharmacotherapy coverage drug policy was shown to be an effective intervention to improve 26-week quit rates in Canada. Medication adherence needs to be promoted by prescribers to enhance quitting. Relapse prevention strategies that include preventing and managing slips through extended behavioural and or pharmacological interventions or combination medication might be required for smokers who respond to pharmacotherapy but relapse once medication is discontinued. Further research is needed to establish the actual duration of coverage required for sustained effects on smoking cessation.

## Competing interests

Pfizer sponsored the study and recruited study sites based on various factors, including but not limited to, clinical research experience and capabilities, smoking cessation expertise/interest, and referral from peers. Data were analyzed by Pfizer and made available to the authors for interpretation and preparation of the manuscript. PS, GB, and PO did not receive honoraria for their participation or for writing the manuscript. VR, CA, and SR are employees of Pfizer Inc. PO, PS, and GB declare financial compensation from Pfizer Inc. for professional services, including protocol and clinical trial materials development, initial start-up, and end-of-study activities such as Case Report Form review, Statistical Analysis Plan review, preparation, participation, and presentation at the Investigator Meeting, and Clinical Study Report review. No payments were made by Pfizer Inc. to PO, PS, or GB for authorship and/or authorship-related activities of this paper. CA, SR, and VR are employees of and shareholders in Pfizer Inc.

## Authors’ contributions

PS, GB, SR, PO, and VR were involved in the design of the study. CA performed the statistical analysis of the data. PS, GB, SR, PO, CA, and VR interpreted the data. PS and GB wrote the first draft of the manuscript and made revisions. PS, GB, SR, PO, CA, and VR critically reviewed the manuscript for important intellectual content and accuracy. All authors have read and approved the final version to be published.

## Pre-publication history

The pre-publication history for this paper can be accessed here:

http://www.biomedcentral.com/1471-2458/14/433/prepub

## Supplementary Material

Additional file 1 **Pre-screening, screening, and randomization procedures.** Flow diagram illustrating the pre-screening, screening and randomization procedures involved in the ACCESSATION study. Click here for file

Additional file 2 **Dosage forms and strengths of smoking cessation pharmacotherapies eligible for reimbursement.** Table showing the eligible pharmacological smoking cessation method(s) prescribed according to the most recent version of the respective product monograph or equivalent. Click here for file

Additional file 3 **Continuous abstinence rate *[weeks 26–39 and 26–52].*** Line graph showing continuous abstinence rates for after withdrawal of coverage eligibility (weeks 26 to 52) for those who had previously had full versus no coverage during the intervention phase (first 26 weeks of the study). Click here for file

Additional file 4 **Assumed smoking cessation pharmacotherapy utilization over 26 weeks.** Plot showing pharmacotherapy utilization in the full versus no coverage groups during the intervention phase (first 26 weeks of the study) (post-hoc analysis). Click here for file
