# Supplementary material for: A pragmatic, randomized, controlled study evaluating the impact of access to smoking cessation pharmacotherapy coverage on the proportion of successful quitters in a Canadian population of smokers motivated to quit (ACCESSATION)
Source: BMC Public Health. 2014 May 7;14:433. doi: 10.1186/1471-2458-14-433 (PMC4022549; doi:10.1186/1471-2458-14-433)
Supplement: Additional file 1 — Pre-screening, screening, and randomization procedures. Flow diagram illustrating the pre-screening, screening and randomization procedures involved in the ACCESSATION study. [file 1471-2458-14-433-S1.pdf]

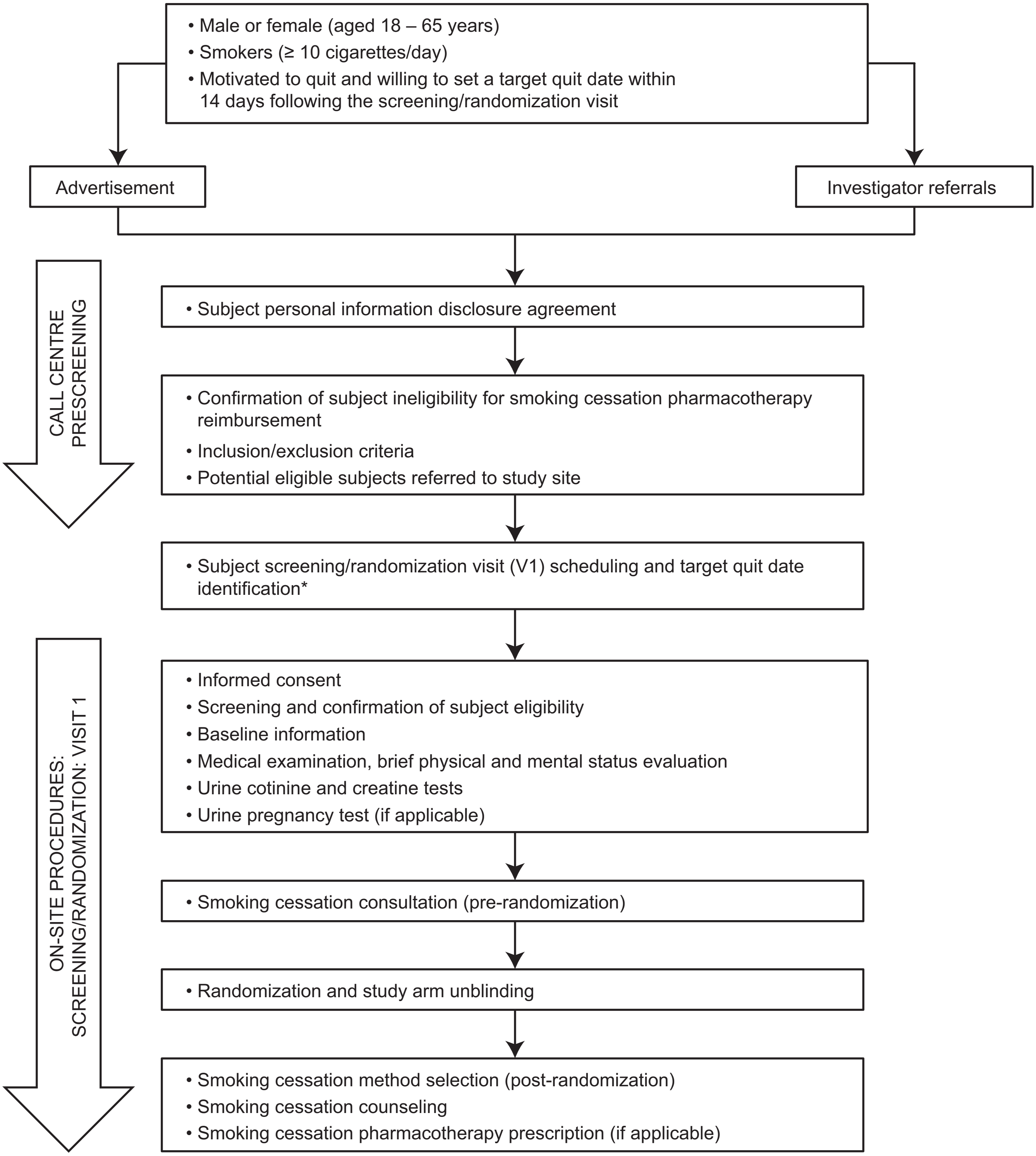

*Subject must have had a target quit date within 14 days following screening/randomization visit

Randomization and study arm unblinding: The study biostatistician provided the randomization scheme to Sampling Technologies Incorporated (STI). The randomization scheme was prepared in advance according to the study arms (1:1 ratio). STI provided SmartPayment™ cards (drug reimbursement cards), which were linked to the specific randomization. Subjects were encouraged to remain with the same local pharmacy for the study duration, but they could change pharmacies, if necessary, STI provided investigators with a blinded lot of randomizations. A randomization code was assigned to each eligible subject. SmartPayment™ cards were contained in sealed envelopes with the randomization codes printed on the envelopes. The study arm (full coverage or no coverage) was blinded to both the investigator and the subject until randomization
