# Supplementary material for: A pragmatic, randomized, controlled study evaluating the impact of access to smoking cessation pharmacotherapy coverage on the proportion of successful quitters in a Canadian population of smokers motivated to quit (ACCESSATION)
Source: BMC Public Health. 2014 May 7;14:433. doi: 10.1186/1471-2458-14-433 (PMC4022549; doi:10.1186/1471-2458-14-433)
Supplement: Additional file 2 — Dosage forms and strengths of smoking cessation pharmacotherapies eligible for reimbursement. Table showing the eligible pharmacological smoking cessation method(s) prescribed according to the most recent version of the respective product monograph or equivalent. [file 1471-2458-14-433-S2.docx]

**Additional file 2**

| Product | Canadian  trade name | Dosage form | Dosage strengths |
| --- | --- | --- | --- |
| Varenicline | Champix | Tablets | 0.5 and 1 mg |
| Bupropion | Zyban | Tablets | 150 mg |
| Nicotine patch | Nicoderm | Patch | 7, 14, and 21 mg/day |
| S(-)-nicotine patch | Habitrol | Patch | 7, 14, and 21 mg/day |
| Nicotine gum | Nicorette | Gum | 2 and 4 mg |
| Nicotine polacrilex gum | Thrive | Gum | 2 and 4 mg |
