# Supplementary material for: A pragmatic, randomized, controlled study evaluating the impact of access to smoking cessation pharmacotherapy coverage on the proportion of successful quitters in a Canadian population of smokers motivated to quit (ACCESSATION)
Source: BMC Public Health. 2014 May 7;14:433. doi: 10.1186/1471-2458-14-433 (PMC4022549; doi:10.1186/1471-2458-14-433)
Supplement: Additional file 3 — Continuous abstinence rate [weeks 26–39 and 26–52]. Line graph showing continuous abstinence rates for after withdrawal of coverage eligibility (weeks 26 to 52) for those who had previously had full versus no coverage during the intervention phase (first 26 weeks of the study). [file 1471-2458-14-433-S3.pdf]

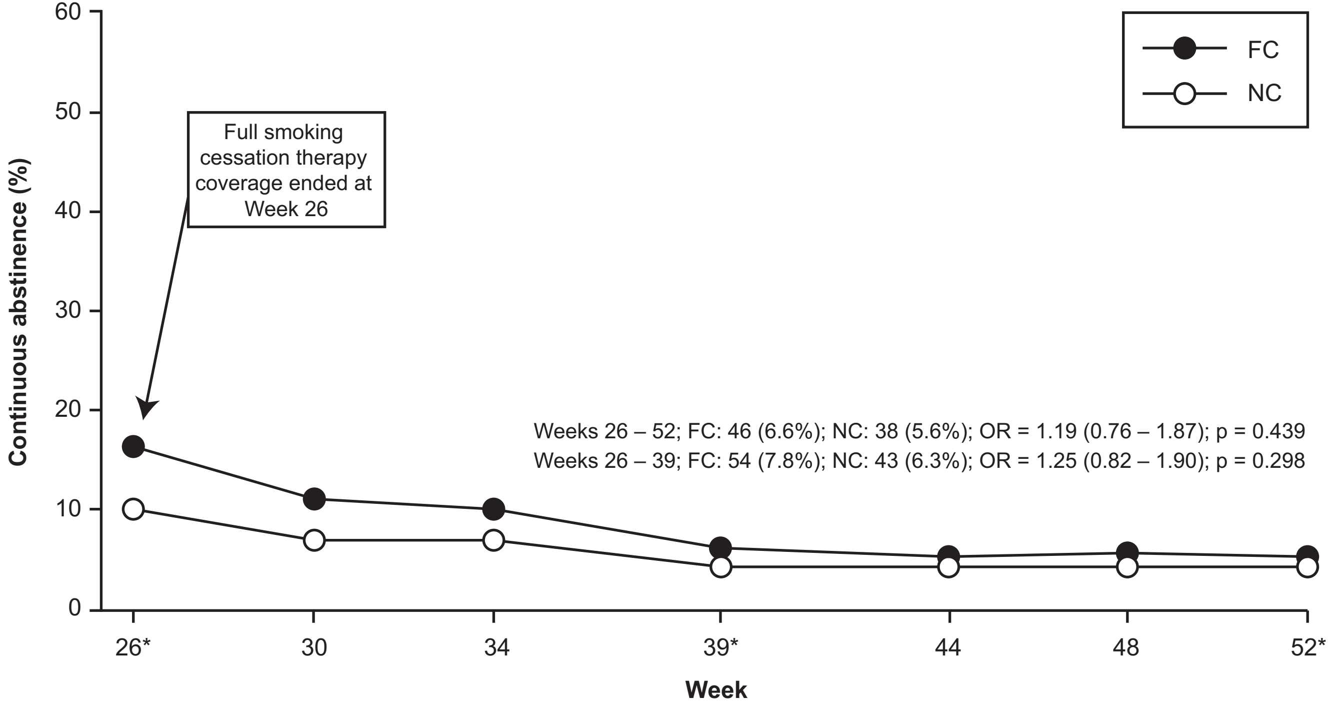

*Clinic visit

Self-reported abstinence confirmed by urine cotinine test at clinic visits

ITT is defined as all randomized subjects

CI = confidence interval; FC = full coverage; ITT = intention-to-treat; NC = no coverage; OR = odds ratio
