# Supplementary material for: A pragmatic, randomized, controlled study evaluating the impact of access to smoking cessation pharmacotherapy coverage on the proportion of successful quitters in a Canadian population of smokers motivated to quit (ACCESSATION)
Source: BMC Public Health. 2014 May 7;14:433. doi: 10.1186/1471-2458-14-433 (PMC4022549; doi:10.1186/1471-2458-14-433)
Supplement: Additional file 4 — Assumed smoking cessation pharmacotherapy utilization over 26 weeks. Plot showing pharmacotherapy utilization in the full versus no coverage groups during the intervention phase (first 26 weeks of the study) (post-hoc analysis). [file 1471-2458-14-433-S4.pdf]

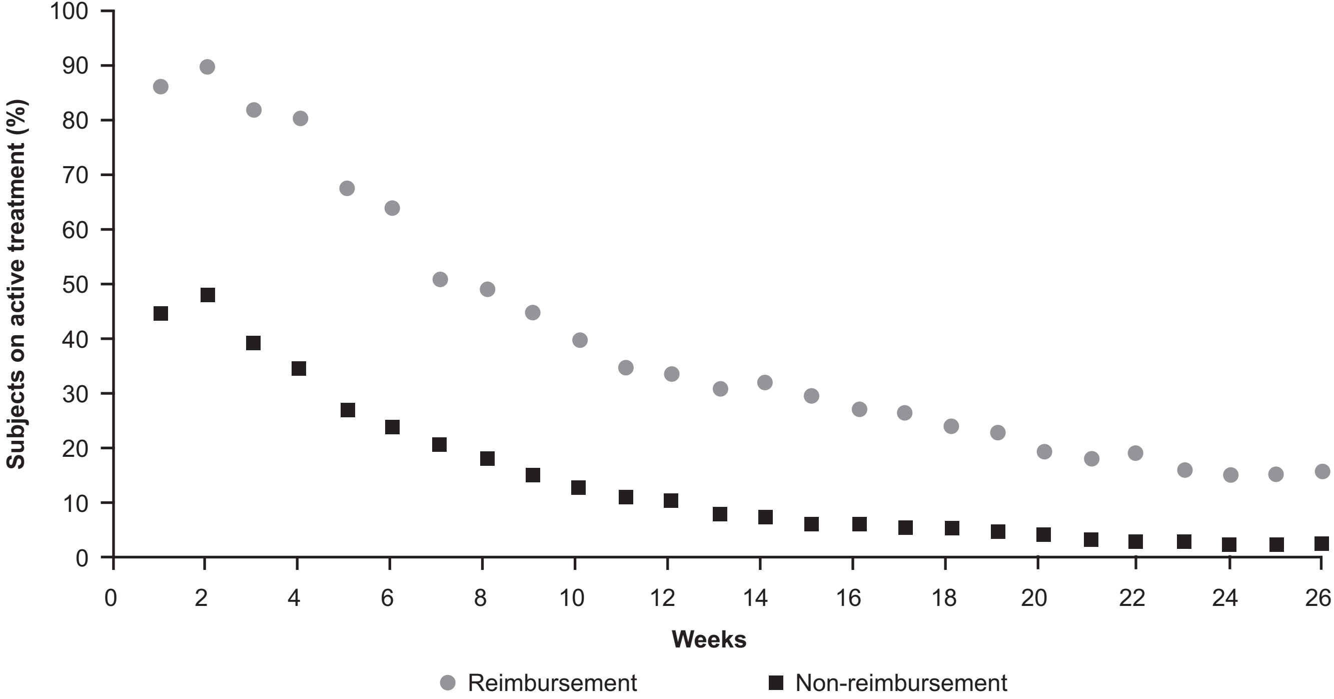

A subject was assumed to be on an active treatment if the number of days elapsed since the last pharmacy service was lower than the number of daily doses supplied at the pharmacy at that service. For instance, if Subject A was dispensed a 4-week supply of varenicline on Week 2 of the study, the subject would be assumed to be on taking varenicline for study Weeks 2 – 5
